# Lysine-specific demethylase 1 mediates epidermal growth factor signaling to promote cell migration in ovarian cancer cells

**DOI:** 10.1038/srep15344

**Published:** 2015-10-22

**Authors:** Genbao Shao, Jie Wang, Yuanxia Li, Xiuwen Liu, Xiaodong Xie, Xiaolei Wan, Meina Yan, Jie Jin, Qiong Lin, Haitao Zhu, Liuping Zhang, Aihua Gong, Qixiang Shao, Chaoyang Wu

**Affiliations:** 1 School of Medicine, Jiangsu University, Zhenjiang 212013, Jiangsu, P. R. China; 2 Department of Oncology, the Affiliated People’s Hospital, Jiangsu University, Zhenjiang 212002, Jiangsu, P. R. China; 3 Jiangsu Key Laboratory of Medical Science and Laboratory Medicine, School of Medicine, Jiangsu University, Zhenjiang 212013, Jiangsu, P. R. China; 4 Department of Radiology, the Affiliated Hospital, Jiangsu University, Zhenjiang 212001, Jiangsu, P. R. China; 5 Gaochun People’s Hospital, Nanjing 211300, Jiangsu, P. R. China

## Abstract

Epigenetic abnormalities play a vital role in the progression of ovarian cancer. Lysine-specific demethylase 1 (LSD1/KDM1A) acts as an epigenetic regulator and is overexpressed in ovarian tumors. However, the upstream regulator of LSD1 expression in this cancer remains elusive. Here, we show that epidermal growth factor (EGF) signaling upregulates LSD1 protein levels in SKOV3 and HO8910 ovarian cancer cells overexpressing both LSD1 and the EGF receptor. This effect is correlated with a decrease in the dimethylation of H3K4, a major substrate of LSD1, in an LSD1-dependent manner. We also show that inhibition of PI3K/AKT, but not MEK, abolishes the EGF-induced upregulation of LSD1 and cell migration, indicating that the PI3K/PDK1/AKT pathway mediates the EGF-induced expression of LSD1 and cell migration. Significantly, LSD1 knockdown or inhibition of LSD1 activity impairs both intrinsic and EGF-induced cell migration in SKOV3 and HO8910 cells. These results highlight a novel mechanism regulating LSD1 expression and identify LSD1 as a promising therapeutic target for treating metastatic ovarian cancer driven by EGF signaling.

Lysine-specific demethylase 1 (LSD1/KDM1A/AOF2/BHC110/KIA0601) is a highly conserved flavin adenine dinucleotide (FAD)-dependent amine oxidase. LSD1 was initially found to specifically remove mono- and dimethyl groups from methylated histone H3 at lysine 4 (H3K4me1/2) to suppress gene expression^1^,^2^. In prostate cancer cells, it also demethylates repressive mono- and dimethylated lysine 9 (H3K9me1/2) in an androgen-receptor-dependent manner^3^. LSD1 is frequently overexpressed in several types of malignancies, including breast^4^, prostate^5^, bladder^6^, lung^6^, colon^7^, neuroblastoma^8^, and hepatocellular cancer^9^. Importantly, overexpression of LSD1 promotes cell proliferation, migration, and invasion in colon, lung, and gastric cancers^10^,^11^,^12^. It also contributes to the oncogenic potential of MLL-AF9 leukemia stem cells and acute myeloid leukemia^13^,^14^. Recently, two studies showed that LSD1 is overexpressed in ovarian cancer tissues and cell lines^15^,^16^, and LSD1 plays an important role in ovarian cancer cell proliferation via a Sox2-mediated mechanism^17^. However, the upstream events that regulate LSD1 expression remain largely unknown.

Epidermal growth factor receptor (EGFR) signaling regulates various developmental events in the ovary, including follicle formation and epithelium cell growth. Dysregulation of EGFR signaling promotes the progression of epithelial ovarian cancer^18^,^19^,^20^. EGFR binding of EGF initiates several signal transduction pathways, including the phosphatidylinositol 3-kinase (PI3K)/AKT and mitogen-activated protein kinase (MAPK)/ERK pathways, and substantially enhances cancer cell motility^21^,^22^. Overexpression of EGFR is associated with an aggressive phenotype^23^,^24^ and poor prognosis of ovarian tumors^25^,^26^. Thus, anticancer agents that target EGFR or its downstream signaling pathways hold great promise for treatment of ovarian cancer.

In this report, we show that LSD1 is overexpressed in SKOV3, HO8910, and 3AO ovarian cancer cells, and its levels increase in parallel with increased levels of EGFR. More importantly, EGF upregulates LSD1 protein levels via activation of the PI3K/AKT pathway, but not the MAPK/ERK pathway. This effect is correlated with an expected decrease in the levels of H3K4me2, a major substrate of LSD1, in an LSD1-dependent manner. Furthermore, upregulation of LSD1 enhances EGF-induced migration of SKOV3 and HO8910 cells. To our knowledge, the findings presented in this report are the first to demonstrate that LSD1 mediates EGFR signaling-dependent ovarian cancer cell migration. The results provided in this report suggest that targeting LSD1 may be an effective approach for inhibiting the progression of ovarian cancer, particularly EGFR signaling-dependent progression.

## Results

### LSD1 expression is elevated in ovarian cancer cells that overexpress EGFR

**Figure 1:**
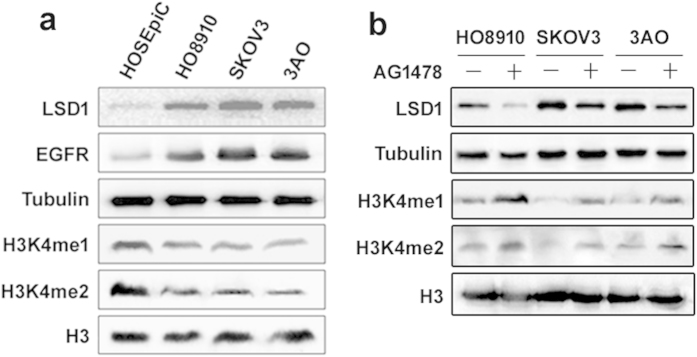
LSD1 expression is associated with sensitivity to EGFR inhibition in ovarian cancer cell lines. (**a**) Protein levels were detected via western blotting in whole-cell extracts (LSD1 and EGFR) or histone protein extracts (H3K4me1 and me2) from ovarian epithelial HOSEpiC cells and HO8910, SKOV3, and 3AO ovarian cancer cells. (**b**) HO8910, SKOV3, and 3AO cells were treated with the EGFR inhibitor AG1478 (10 μM) for 24 h, after which the levels of LSD1 protein and H3K4 methylation were analyzed via western blotting. α-tubulin and histone H3: loading control.

To determine the relationship between LSD1 and EGFR, we analyzed the protein expression of LSD1 and EGFR in the SKOV3, HO8910, and 3AO ovarian cancer cell lines. Increased expression of LSD1 and decreased levels of its substrates H3K4me1 and me2 were noted in the three cell lines (Fig. 1a). Similarly, increased EGFR expression was detected in these cells (Fig. 1a). The observed changes in LSD1 and H3K4me1/2 expression were reversed in all three cell lines in response to an EGFR inhibitor (Fig. 1b), suggesting that there is cross-talk between the LSD1 and EGFR pathways.

### EGF increases LSD1 levels in ovarian cancer cells

**Figure 2:**
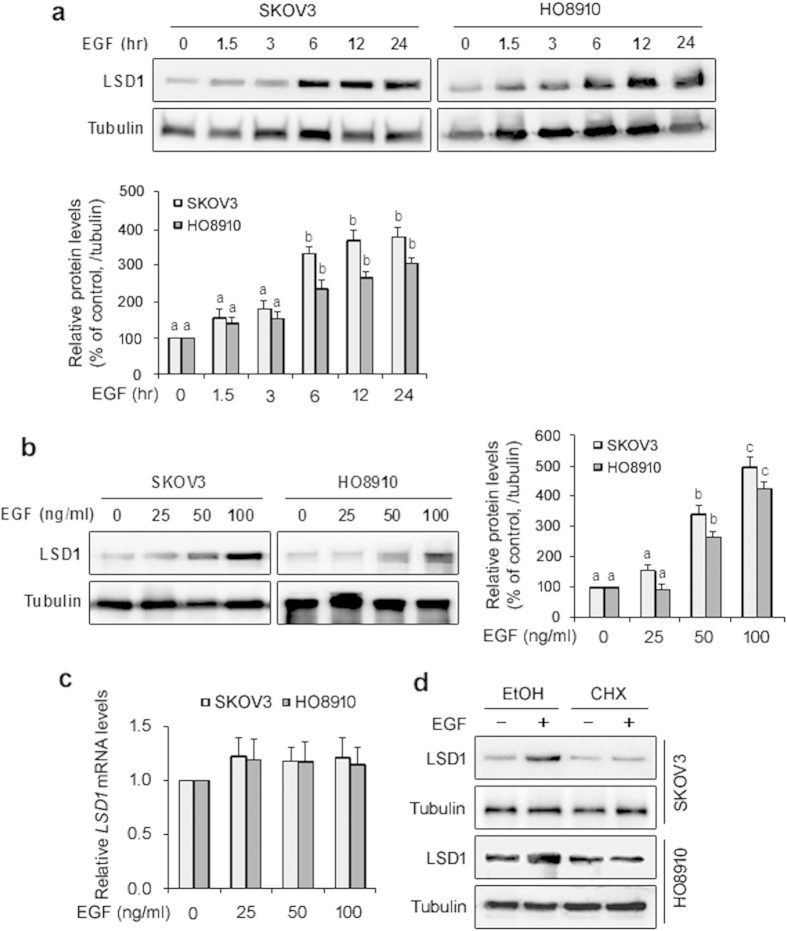
EGF stimulates upregulation of the LSD1 protein in SKOV3 and HO8910 cells. (**a**) SKOV3 and HO8910 cells were treated with 100 ng/ml EGF for the indicated times, and the induced expression of the LSD1 protein was monitored and quantified through western blotting. α-tubulin was used as a loading control. The error bars represent the mean ± SEM (n = 3). (**b**) SKOV3 and HO8910 cells were treated with different doses of EGF for 24 h, after which LSD1 protein levels were detected via western blotting. α-tubulin was used as a loading control. The error bars represent the mean ± SEM (n = 3). Values within the same row with different superscripted letters are significantly different, *p* < 0.05 (one-way ANOVA). (**c**) SKOV3 and HO8910 cells were treated with different doses of EGF for 12 h, after which LSD1 mRNA levels were detected via Q-PCR. The transcript levels of the *LSD1* gene were normalized against those of *GAPDH*, and the value for the untreated control (0 ng/ml EGF) was set to 1. The error bars represent the mean ± SEM (n = 4). (**d**) SKOV3 and HO8910 cells were pretreated with 10 μg/ml cycloheximide (CHX) for 1 h before the addition of EGF (100 ng/ml) for 24 h, after which the cells were harvested for immunoblotting analysis of LSD1. Ethanol (EtOH) was included as a vehicle control.

To confirm the functional relationship between LSD1 and EGFR, we investigated the effect of EGF on LSD1 expression in the SKOV3 and HO8910 cell lines. Our results indicated that treatment with EGF upregulated LSD1 protein levels in a time-dependent manner (Fig. 2a). EGF treatment also caused a dose-dependent increase in LSD1 protein levels (Fig. 2b). However, EGF treatment did not significantly alter *LSD1* mRNA levels (Fig. 2c). The increase in LSD1 protein levels in response to EGF was blocked by CHX (Fig. 2d), an inhibitor of protein synthesis, suggesting that the EGF-induced increase in LSD1 occurs at the translational level because no changes in *LSD1* mRNA levels were observed (Fig. 2c).

**Figure 3:**
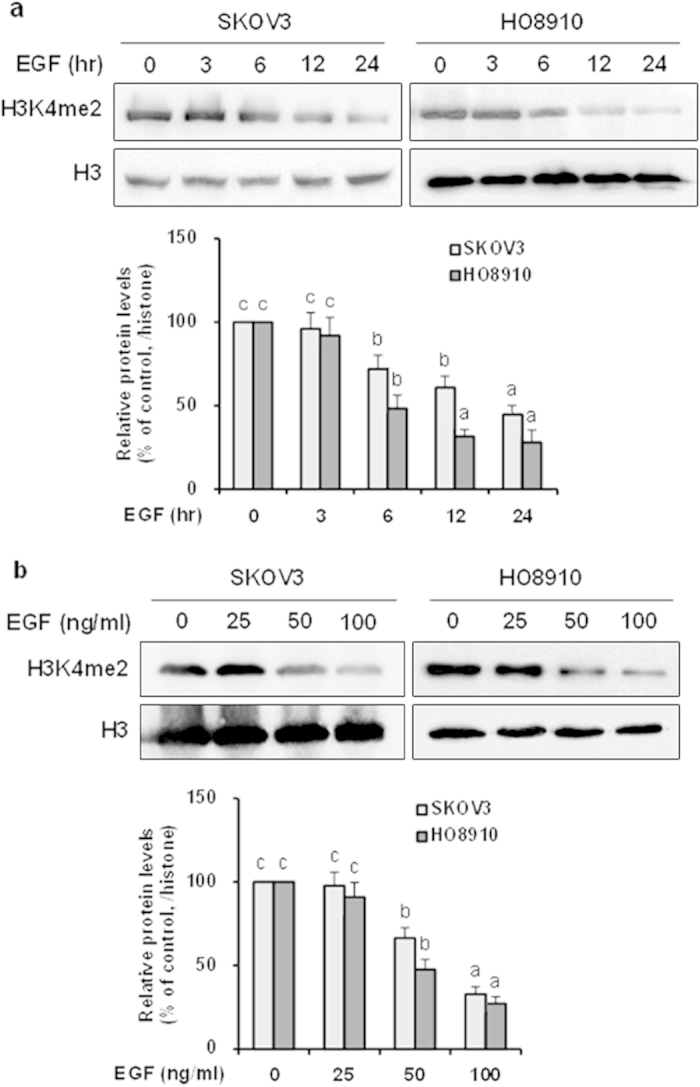
EGF suppresses the levels of H3K4me2 in SKOV3 and HO8910 cells. (**a**) SKOV3 and HO8910 cells were treated with 100 ng/ml EGF for the indicated times. Histone protein extracts harvested from the treated cells were subjected to western blotting and probed for H3K4me2 and histone H3. The error bars represent the mean ± SEM (n = 3). (**b**) SKOV3 and HO8910 cells were treated with different doses of EGF for 24 h, after which the global levels of H3K4me2 and histone H3 were analyzed via western blotting. The error bars represent the mean ± SEM (n = 3). Values within the same row with different superscripted letters are significantly different, *p* < 0.05 (one-way ANOVA).

**Figure 4:**
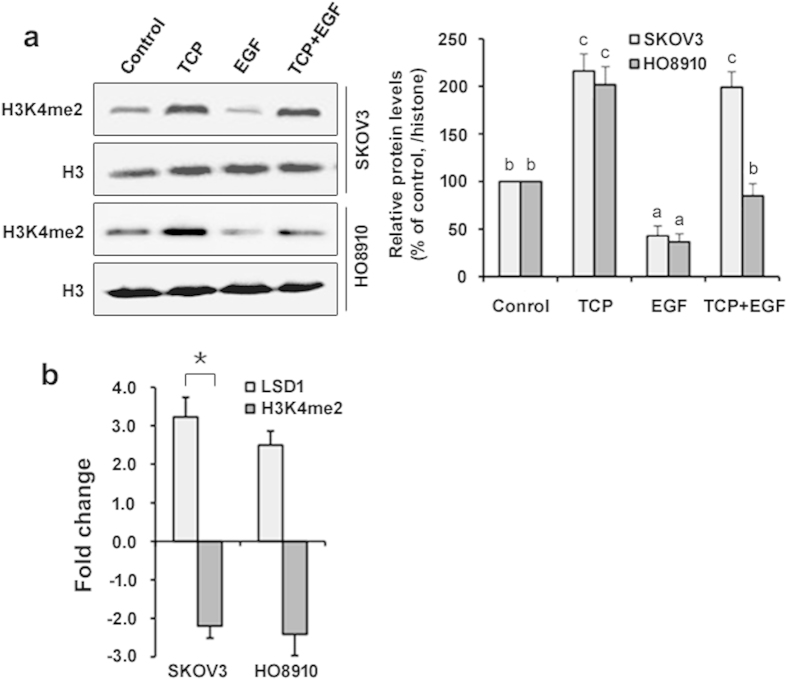
Inactivation of LSD1 reverses the EGF-induced reduction of H3K4me2. (**a**) SKOV3 and HO8910 cells were treated with the LSD1 inhibitor tranylcypromine (TCP, 100 μM) either alone or in the presence of 100 ng/ml EGF for 24 h. Histones isolated from the treated cells were subjected to immunoblotting for H3K4me2 and histone H3. The error bars represent the mean ± SEM (n = 3). Values within the same row with different superscripted letters are significantly different, *p* < 0.05 (one-way ANOVA). (**b**) SKOV3 and HO8910 cells were treated with 100 ng/ml EGF for 24 h. The levels of LSD1 and H3K4me2 were monitored via western blotting with specific antibodies. α-tubulin and histone H3: loading control. The relative levels were calculated as the fold change in the expression of LSD1, while the fold change in the demethylase activity of LSD1 was calculated using the following formula: 1/the relative levels of H3K4me2. The error bars represent the mean ± SEM (n = 3). The data were analyzed with Student’s *t-*test. ^*^p < 0.05.

The fact that LSD1 is markedly upregulated by EGF suggested that a reduction of H3K4 methylation may be observed upon EGF stimulation. Consistent with this prediction, we found that H3K4me2 was significantly decreased in both cell lines upon EGF treatment in a time- and dose-dependent fashion (Fig. 3a,b). To determine whether the decrease in H3K4me2 was dependent on LSD1, we used a potent inhibitor, TCP^14^,^17^,^27^, to suppress the demethylase activity of LSD1 in both cell lines in the presence or absence of EGF. The decrease in H3K4me2 observed in response to EGF was completely blocked by TCP in SKOV3 cells and partially blocked in HO8910 cells (*P* < 0.05; Fig. 4a). These data suggest that LSD1 is a major player that mediates H3K4me2 demethylation during EGF stimulation, although the possible involvement of another H3K4 demethylase in this process cannot be excluded.

Next, we compared the fold changes in the expression levels of LSD1 and its substrate H3K4me2 in response to EGF. There was a significantly greater fold change in LSD1 compared with H3K4me2 in SKOV3 cells (3.2 vs. 2.2; *p* < 0.05), but not in HO8910 cells (2.5 vs. 2.4; Fig. 4b), suggesting that EGF plays a major role in the regulation of LSD1 expression. Thus, our data indicate that the reduction of H3K4me2 is likely a direct consequence of LSD1 upregulation which in turn is in response to EGF.

### The PI3K/AKT pathway mediates the regulation of LSD1 in ovarian cancer cells

**Figure 5:**
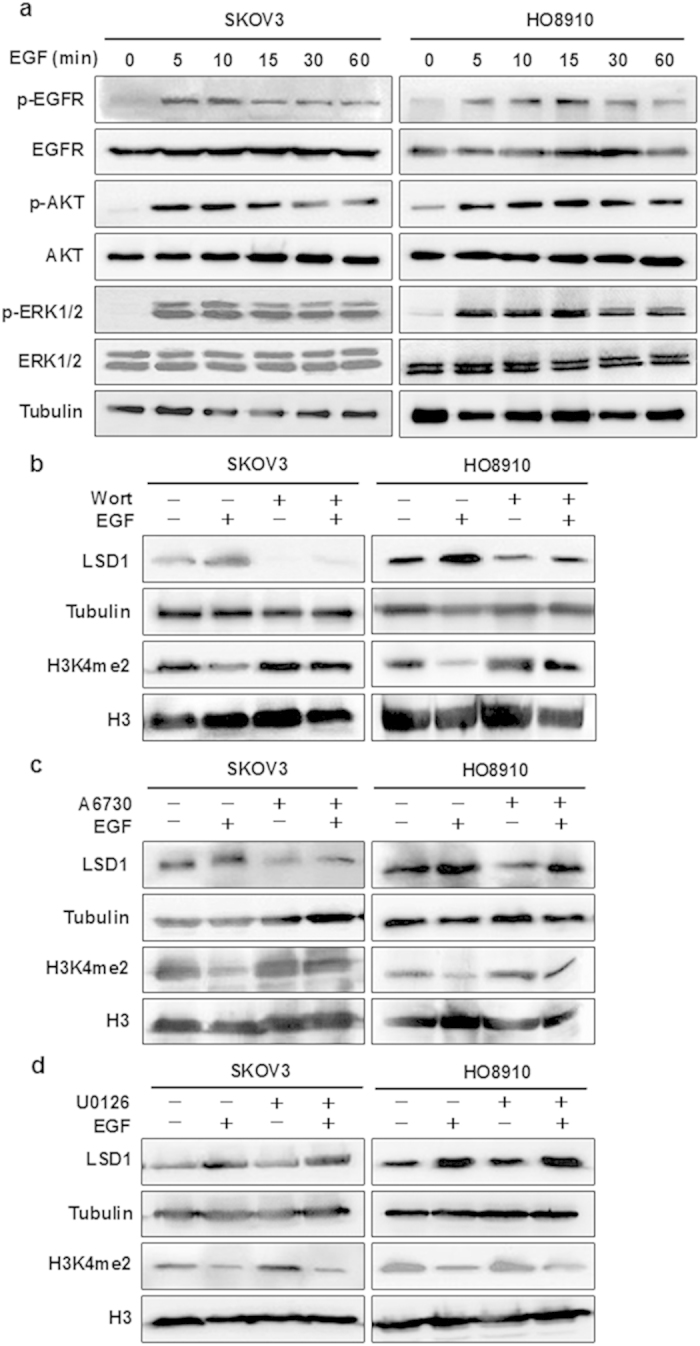
EGF elevates LSD1 expression via the PI3K/AKT pathway. (**a**) SKOV3 and HO8910 cells were treated with 100 ng/ml EGF for the indicated times. The harvested cells were then subjected to western blotting and probed for phosphorylated EGFR (Tyr992) and total EGFR, phosphorylated AKT (Ser473) and total AKT, and phosphorylated ERK1/2 (Thr202/Tyr204) and total ERK1/2. α-tubulin was used as a loading control. (**b**–**d**) SKOV3 and HO8910 cells were pretreated with the PI3K inhibitor wortmannin (1 μM, **b**), the AKT inhibitor A6730 (20 μM, **c**), or the MEK inhibitor U0126 (10 μM, **d**) for 30 min prior to the addition of EGF (100 ng/ml) for 24 h. Protein levels were detected via western blotting in whole-cell extracts (LSD1) or histone protein extracts (H3K4me2) from the above treated cells. α-tubulin and histone H3: loading control.

We next wished to determine the mechanism by which EGF increases LSD1 expression. We first examined the phosphorylation status of EGFR and its two major downstream effectors, AKT and ERK, in response to EGF stimulation. We found that EGF treatment induced the phosphorylation of EGFR (Tyr992), AKT (Ser473), and ERK (Thr202/Tyr204) in a time-dependent manner in SKOV3 and HO8910 cells (Fig. 5a). We then examined whether these two pathways are involved in the upregulation of LSD1 expression by EGF. The PI3K/AKT pathway was specifically inhibited using wortmannin or A6730^28^,^29^, and the MAPK/ERK pathway was inhibited with U0126^22^,^30^. Treatment with wortmannin or A6730 decreased basal LSD1 expression and partially abolished the EGF-induced upregulation of LSD1 expression (Fig. 5b,c). The effect of both inhibitors on LSD1 expression was consistent with the increase in global levels of H3K4me2 (Fig. 5b,c). In contrast, U0126 treatment had no impact on the levels of LSD1 and H3K4me2 in either cell line (Fig. 5d). These results indicate that the PI3K/AKT pathway, but not the MAPK/ERK pathway, mediates the EGF-induced upregulation of LSD1.

### LSD1 is involved in EGF-induced ovarian cancer cell migration

**Figure 6:**
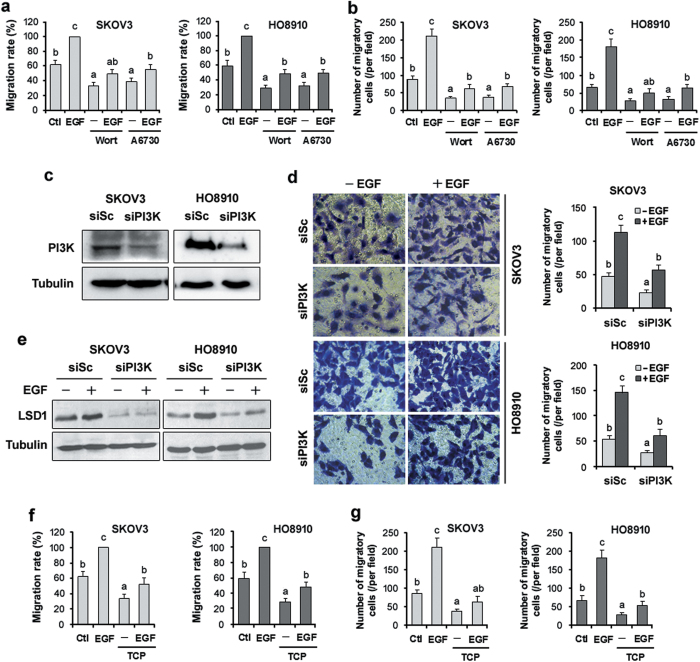
Inhibition of LSD1 reduces EGF-induced cell migration. (**a**,**b**) SKOV3 and HO8910 cells were pretreated with wortmannin (1 μM) or A6730 (20 μM) for 30 min and then stimulated with 100 ng/ml EGF in the presence of the inhibitors for 24 h. The migration of both cell lines was analyzed in wound-healing (**a**) and Transwell assays (**b**). Representative images are presented in Supplementary Figures S1 and S2. (**c**) SKOV3 and HO8910 cells were transfected with scrambled siRNA (siSc) or siRNA specific for PI3K (siPI3K) for 72 h. The protein levels of PI3K were determined via western blot analysis. (**d**) After 72 h of transfection, trypsinized cells were seeded in Transwell inserts and cultured with 100 ng/ml EGF for 24 h. The left panels show representative images of migrated cells (40 × magnification), and the right panels show the quantification of the average number of migrated cells per well. (**e**) The expression of LSD1 was detected in the transfected cells via western blotting. α-tubulin: loading control. (**f**,**g**) SKOV3 and HO8910 cells were pretreated with TCP (100 μM) for 30 min and then cotreated with 100 ng/ml EGF for 24 h. The migration of both cell lines was analyzed via wound-healing (f) and Transwell assays (**g**). Representative images are presented in Supplementary Figures S1 and S2. The error bars represent the mean ± SEM [(**a**,**b**,**d**) n = 3; (**f**,**g**) n = 4; values within the same row with different superscripted letters are significantly different, *p* < 0.05]. The data were analyzed through one-way ANOVA followed by Tukey’s multiple comparison test.

Previous observations that EGF enhances ovarian cancer cell migration^22^,^31^ prompted us to test whether LSD1 was involved in this process. We first assessed the involvement of the PI3K/AKT signaling pathway in EGF-induced cell migration. SKOV3 and HO8910 cells treated with either the PI3K inhibitor wortmannin or the AKT inhibitor A6730 displayed decreased basal migration and significantly reduced EGF-induced cell migration in wound-healing (Fig. 6a and Fig. S1?) and Transwell assays (Fig. 6b and Fig. S2). We further determined the effect of PI3K signaling on cell migration through knockdown of PI3K. We observed that transfection of SKOV3 and HO8910 cells with siRNA specific for the PI3K catalytic subunit p110α decreased the levels of this protein (Fig. 6c). The underexpression of PI3K significantly diminished EGF-induced cell migration (Fig. 6d). Knockdown of PI3K also reduced the EGF-induced expression of LSD1 (Fig. 6e). This result agrees with the finding that inhibition of PI3K by wortmannin diminished EGF-induced LSD1 expression, as shown in Fig. 5b. These data indicate that the PI3K/AKT pathway is involved in the EGF-induced migration of SKOV3 and HO8910 cells.

**Figure 7:**
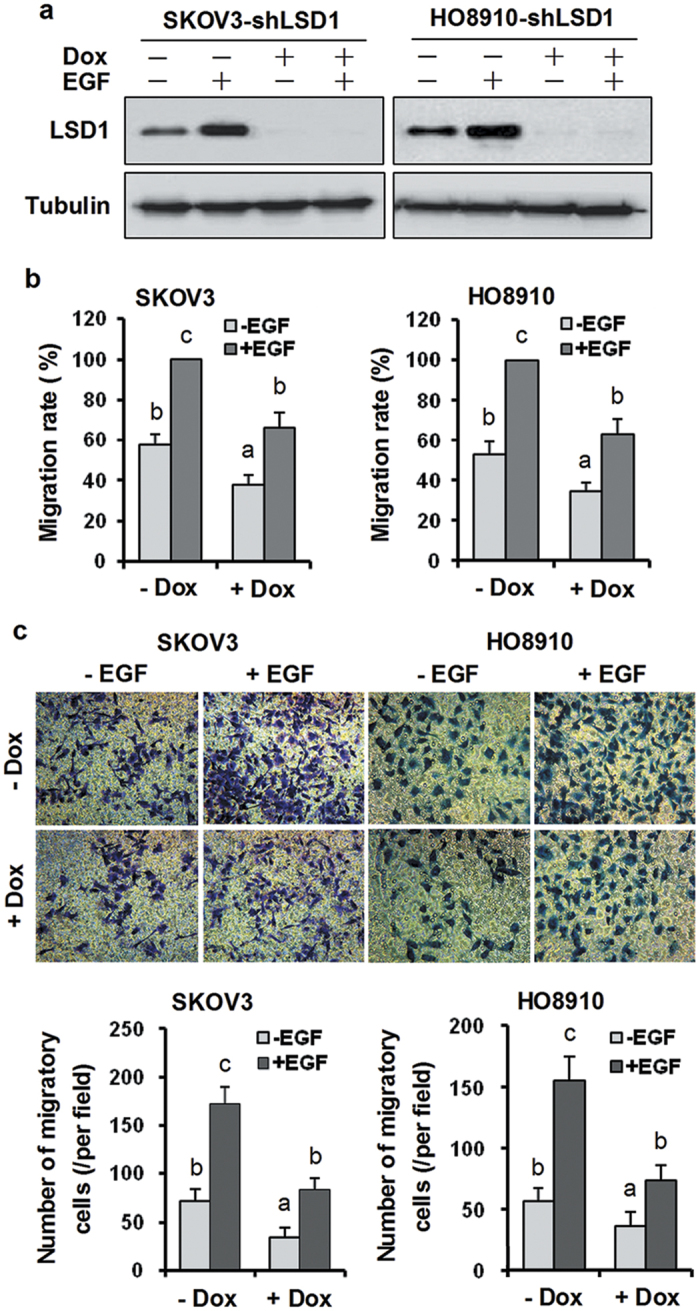
Upregulation of LSD1 is required for EGF-induced cell migration. (**a**) SKOV3 and HO8910 cells were stably transfected with an LSD1 shRNA and then treated with or without 100 ng/ml Dox for 24 h. After 24 h, the cells were treated with 100 ng/ml EGF in the presence of Dox for another 24 h and subjected to immunoblotting for LSD1 and α-tubulin. (**b**) Twenty-four hours post Dox treatment, the cells were scratched using a 10-μl pipette tip and subsequently stimulated with 100 ng/ml EGF in the presence of Dox for another 24 h, followed by a wound-healing assay. (**c**) After 24 h of Dox treatment, the trypsinized cells were seeded in Transwell inserts and cultured with 100 ng/ml EGF in the presence of Dox for 24 h. Top panels show representative images (400 × magnification), and the bottom panels provide a graphical representation of the accumulated number of migrated cells at 24 h for both cell lines. The migrated cells were counted in five randomly selected fields within each membrane, and the values were averaged. The error bars represent the mean ± SEM [(**b**,**c**) n = 3; values within the same row with different superscripted letters are significantly different, *p* < 0.05]. The data were analyzed through one-way ANOVA followed by Tukey’s multiple comparison test.

We next confirmed the requirement for LSD1 in EGF-induced cell migration. In the presence of the LSD1 inhibitor TCP, SKOV3 and HO8910 cells exhibited less migration, and cell migration in response to EGF was markedly reduced in wound-healing (Fig. 6f and Fig. S1) and Transwell assays (Fig. 6g and Fig. S2). To further verify the effect of the inhibitor on cell migration, we employed lentiviral shRNA constructs to inducibly knock down LSD1 using Dox in both cell lines (Fig. 7a). These cells were then treated with EGF, and cell migration was assessed in wound-healing and Transwell assays. Consistent with data shown in Fig. 6f,g, knockdown of LSD1 significantly reduced intrinsic migration and totally abolished EGF-induced cell migration (Fig. 7b,c). Collectively, these results suggest that LSD1 plays a role in cell migration and that EGF-induced cell migration requires LSD1 upregulation and PI3K/AKT activation.

## Discussion

Previous studies have confirmed that LSD1 is overexpressed in ovarian tumors^16^,^17^. However, little information exists on the regulation of LSD1 expression in this cancer. In this study, we show that EGF induces upregulation of LSD1, with a concomitant reduction of its substrate H3K4me2, which mediates the EGF-induced migration of SKOV3 and HO8910 ovarian cancer cells. We demonstrate that EGF exerts its effects on LSD1 upregulation and cell migration via activation of the PI3K/PDK1/AKT signaling pathway, which provides a novel mechanism regulating LSD1 expression, leading to cell migration upon EGF stimulation.

LSD1 has been implicated in several types of cancer and linked to cellular growth pathways^32^. In bladder carcinogenesis, LSD1 is overexpressed in tumors even at an early grade^6^, suggesting that LSD1 is one of the initiators of this whole process. LSD1 is also upregulated in poorly differentiated neuroblastomas and is associated with an adverse clinical phenotype^8^. In fact, our observation that the three ovarian cancer cell lines tested show robust levels of the LSD1 protein may also suggest a functional role of LSD1 in these cells. Interestingly, these cell lines showing high levels of LSD1 exhibit a signature of EGFR overexpression as well, suggesting a functional link between LSD1 and the EGFR pathway. Indeed, a recent study found that LSD1 expression was increased by transforming growth factor beta (TGF-β), an EGFR-like signaling pathway, in AML12 cells^33^. Our current data demonstrated that EGF could upregulate the production of LSD1 through activation of the PI3K/AKT pathway in SKOV3 and HO8910 cells. This effect was correlated with a decrease of H3K4me2, a methylated substrate of LSD1. Strikingly, inhibition of LSD1 partially abolished the suppressive effect of EGF on H3K4me2 in HO8910 cells, implying that other H3K4 demethylases may be involved in this process, in addition to LSD1. It has been well documented that AKT signaling can regulate the initiation of translation in response to various growth factors^34^,^35^. Further work is required to elucidate the molecular mechanism by which LSD1 expression is regulated by the AKT pathway. Recently, it was reported that LSD1 is recruited by the nuclear receptor TLX to the promoter of PTEN to repress the expression of the PTEN gene^36^,^37^, which is a known negative regulator of the PI3K/AKT pathway^38^. LSD1 and AKT appear to exhibit dual functions as both cargo and regulators of this pathway, and reciprocal regulation therefore exists. Taken together, our results indicate that EGF-dependent PI3K/AKT activation may contribute to the upregulation of LSD1 in ovarian cancer.

LSD1 was recently shown to be highly expressed in human ovarian cancer tissues and cell lines^15^,^17^, but the function of LSD1 was not investigated. Our data show that upregulation of LSD1 enhances the migration of SKOV3 and HO8910 cells in response to EGF. Conversely, McDonald *et al.* observed that loss of LSD1 function inhibits cell migration in AML12 cells upon TGF-β stimulation^33^. Importantly, we demonstrated that LSD1 depletion impairs intrinsic migration and markedly blocks EGF-induced cell migration. In accordance with our data, LSD1 has been found to promote cell motility in other types of cancer by regulating the expression of migration proteins and migration pathway proteins through epigenetic changes^11^,^39^,^40^. Our findings reveal LSD1 as a promising therapeutic target for preventing the metastasis of ovarian cancer, especially EGF signaling-dependent metastasis. The role of LSD1 in ovarian cancer that is described herein differs from that in breast cancer cells, as LSD1 was reported to inhibit the invasion of breast cancer cells *in vitro* and suppress the metastatic potential of breast cancer *in vivo*^41^. It will be interesting to evaluate the diverse roles of LSD1 in distinct types of cancer.

**Figure 8:**
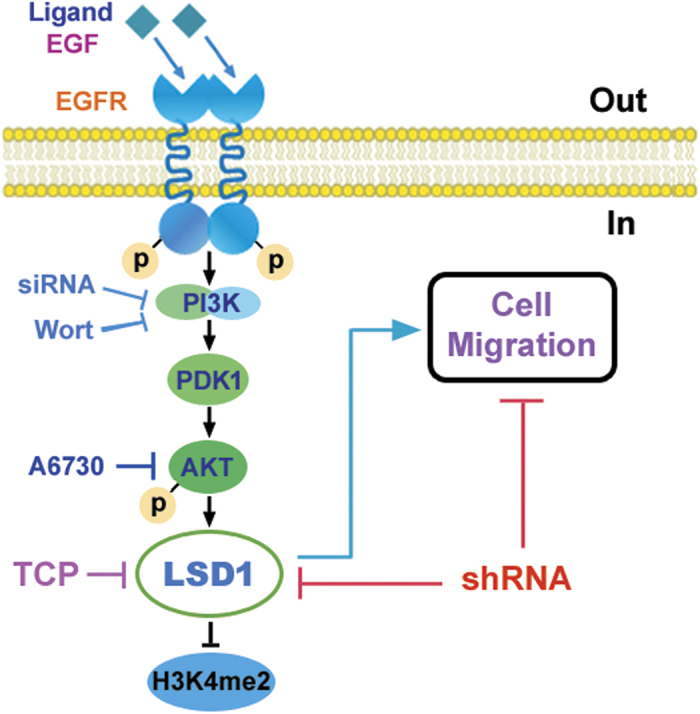
Schematic representation of the pathways required for LSD1 to play its role in EGF-induced cell migration. PI3K transmits EGF/EGFR signals to the downstream effector AKT (as indicated through use of the PI3K antagonist wortmannin, siRNA targeting the p110α catalytic subunit of PI3K, or the AKT antagonist A6730), leading to a decrease in H3K4 methylation and upregulation of the LSD1 protein, which mediates EGF-induced cell migration. Targeting of LSD1 either via silencing of its mRNA or through inhibition of its demethylase activity attenuates the EGF-induced effects. Approaches that disrupt LSD1 function may be effective for inhibiting the progression of EGF-driven tumors.

In summary, these data, which are summarized in Fig. 8, indicate that EGF upregulates the LSD1 protein, which in turn demethylates H3K4me2, through activation of the PI3K/PDK1/AKT pathway. Furthermore, upregulation of LSD1 mediates the EGF-induced migration of SKOV3 and HO8910 cells. Inhibition of PI3K/AKT signaling or knockdown of LSD1 expression attenuates the effects induced by EGF. Our results provide a functional link between EGF signaling and epigenetic regulation through the action of LSD1.

## Methods

### Cell lines and cell culture

A human ovarian surface epithelial cell line (HOSEpiC) was obtained from ScienCell Research Laboratories (Carlsbad, CA, USA), and three commercially available ovarian epithelial cancer cell lines, SKOV3, HO8910, and 3AO, were generously provided by Dr. Qixiang Shao of Jiangsu University (Zhenjiang, China). SKOV3 was derived from the ascites of a 64-year-old Caucasian female. HO8910 and 3AO were derived from the ascites of Chinese patients with ovarian serous adenocarcinomas. The cells were cultured in McCoy’s 5A medium (SKOV3; Sigma-Aldrich, St. Louis, MO, USA) or RPMI 1640 medium (HOSEpiC, HO8910, and 3AO; Gibco, Grand Island, NY, USA) with 10% fetal bovine serum (FBS, Gibco) at a temperature of 37 °C under 5% CO_2_. HEK 293T cells were cultured in Dulbecco’s modified Eagle’s medium (Gibco) containing 10% FBS at a temperature of 37 °C under 5% CO_2_.

### Antibodies and reagents

The pLKO-Tet-On, pHR’-CMV-8.2ΔVPR, and pHR’-CMV-VSVG vectors were kind gifts from Dr. Changdeng Hu (Purdue University, West Lafayette, IN, USA). LSD1, EGFR, phospho-EGFR (Tyr992), AKT, phospho-AKT (Ser473), p44/42 MAPK (ERK1/2), and phospho-p44/42 MAPK (Thr202/Tyr204) antibodies were purchased from Cell Signaling Technology (Danvers, MA, USA). PI3K p110α, α-tubulin, and the Bioepitope Nuclear and Cytoplasmic Extraction Kit were obtained from Bioworld Technology (St. Louis Park, MN, USA). H3K4me1 and me2 antibodies were purchased from Upstate Biotechnology (Lake Placid, NY, USA). A histone H3 antibody was obtained from Abcam (Cambridge, MA, USA) and Millipore (Billerica, MA, USA). Horseradish peroxidase (HRP)-conjugated secondary antibodies were procured from Jackson Immuno Research Laboratories (West Grove, PA, USA). Electrochemiluminescence (ECL) reagents were purchased from Millipore. Human EGF was obtained from Invitrogen (Carlsbad, CA, USA). The EGFR inhibitor AG1478, the PI3K inhibitor wortmannin, the AKT inhibitor A6730, the MEK inhibitor U0126, and polybrene, doxycycline (Dox), cycloheximide (CHX), and mytomycin C were procured from Sigma-Aldrich, and the LSD1 inhibitor tranylcypromine (TCP) was obtained from Biomol International (Plymouth Meeting, PA, USA). The chemicals were dissolved in water for TCP (137 mM), in dimethyl sulfoxide (DMSO) for A6730 (20 mM) and U0126 (10 mM), or in ethanol for CHX (10 mg/ml).

### Plasmid constructs and transfections

To generate the LSD1 short hairpin RNA (shRNA) construct, annealed short hairpin oligonucleotides (RNAi Consortium collection TRCN0000046072; Sigma-Aldrich) targeting CCACGAGTCAAACCTTTATTT in the coding regions (CDS) of LSD1 were cloned into pLKO-Tet-On using the AgeI and EcoRI sites to produce pLKO-Tet-On-shLSD1. Scrambled siRNA and siRNA for PI3K p110α were obtained from Santa Cruz Biotechnology (Santa Cruz, CA, USA).

All transfections were performed using the Lipofectamine 2000 reagent (Invitrogen) or a siRNA transfection reagent (Santa Cruz Biotechnology) according to the manufacturer’s protocol.

### Generation of stable LSD1 knockdown cell lines

To generate lentiviral particles, HEK 293T cells cultured in 100 mm culture dishes were cotransfected with 2 μg of pLKO-Tet-On-shLSD1, 1.5 μg of pHR’-CMV-8.2ΔVPR, and 0.5 μg of pHR’-CMV-VSVG using the Lipofectamine 2000 reagent (Invitrogen). The supernatant containing lentivirus was harvested at 48 and 72 h posttransfection, then clarified via filtration through a 0.45-μm membrane filter (Millipore), and concentrated via ultracentrifugation at 70,000 × g for 2 h at 4 °C (Avanti^TM^ J-30I, Beckman Coulter, Brea, CA, USA). Infection was performed by adding 1 ml of the lentiviral supernatant to SKOV3 and HO8910 cells in a 60 mm culture dish with 4 ml of growth medium supplemented with 8 μg/ml polybrene (Sigma-Aldrich). Two days after infection, the cells were selected with 1.5 μg/ml puromycin (Invitrogen) for one week until stable clones were established, and the stable clones were confirmed through western blot analysis^42^.

### Small interfering RNA (siRNA) knockdown

SKOV3 or HO8910 cells seeded in a 6-well plate were transfected with a PI3K p110α-specific siRNA or scrambled siRNA at a 50 nM concentration in the presence of a siRNA transfection reagent (Santa Cruz Biotechnology), according to the manufacturer’s instructions. After 7 h, the cells were replenished with 1 ml of normal growth medium containing 20% FBS without removing the transfection mixture. After an additional 24 h of incubation, the medium was replaced with fresh normal growth medium and cultured for a further 48 h and harvested for western blot analysis.

### RNA extraction and quantitative real-time PCR

Total RNA extraction and cDNA synthesis were performed as described previously^43^. Briefly, total RNA was extracted using the TRIzol reagent (Invitrogen), followed by treatment with DNase I (Takara, Shiga, Japan), and 2 μg of RNA was reverse-transcribed using the PrimeScript RT Reagent Kit (Takara) according to the manufacturer’s instructions.

All gene transcripts were quantified via quantitative real-time polymerase chain reaction (Q-PCR). The primer sequences used in the present study were as follows: *LSD1* (GenBank accession number NM_015013.3), 5′-CAAGTGTCAATTTGTTCGGG-3′ (forward) and 5′-TTCTTTGGGCTGAGGTACTG-3′ (reverse); and *GAPDH* (GenBank accession number NM_001256799.1), 5′-GCAAATTCCATGGCACCGTC-3′ (forward) and 5′-TCGCCCCACTTGATTTTGG-3′ (reverse). All PCR assays were performed in a Bio-Rad CFX96 system with SsoFast EvaGreen Supermix (Bio-Rad, Hercules, CA, USA) according to the manufacturer’s instructions. To identify the specific amplification of a single PCR product, the product was confirmed through 2% agarose gel electrophoresis. Negative controls, consisting of the PCR reaction mixture without nucleic acid, were also run with each group of samples. Relative quantification of mRNA levels was performed using the comparative cycle threshold method (2^−ΔΔCT^) with *GAPDH* as the reference gene^43^.

### Histone protein and whole-cell extraction and western blot analysis

Histones were prepared using the Bioepitope Nuclear and Cytoplasmic Extraction Kit (Bioworld Technology) following the manufacturer’s protocol. Briefly, the cells reached 80% to 90% confluence in a 100 mm culture dish prior to collection. The cell nuclei were extracted in 800 μl of reagent A supplemented with complete protease inhibitor cocktail tablets (Roche, Indianapolis, IN, USA). Nuclear lysates were transferred to 1.5 ml centrifuge tubes and placed on ice for 20 min, during which they were vortexed for 15 s every 5 min to resuspend the precipitate. Nuclear pellets were obtained via centrifugation at 3000 × g at 4 °C for 10 min, and the supernatant was collected to obtain cytoplasmic protein. Histone proteins were extracted through resuspension of the nuclear pellets in 150 μl of reagent B with protease inhibitors and incubation on ice for 10 min, after which they were vortexed for 15 s every 5 min to resuspend the precipitate, for a total incubation time of 20 min. After centrifugation at 12,000 × g at 4 °C for 20 min, the supernatant containing histone proteins was collected. Total cellular proteins were isolated directly from cultures in 100 mm Petri dishes after being washed with ice-cold PBS and the addition of 200 μl Cell and Tissue Protein Extraction Reagent (Kangchen Biotech, Shanghai, China), containing protease inhibitor and phosphatase inhibitor cocktails (Roche). The protein extracts were then collected after 20 min lysis on ice and centrifuged for 14,000 × g at 4 °C for 15 min.

The protein concentration was determined using the BCA Protein Assay (Kangchen Biotech). Forty micrograms of total protein and 8 μg of histones were separated on 8–12% SDS-PAGE gels and transferred to polyvinylidene difluoride membranes (Bio-Rad). The membranes were blocked with 5% milk/TBS-T (0.1% Tween-20) for 1 h and immunoprobed with an antibody (diluted in 5% BSA/TBS-T) against LSD1 (1:1000), EGFR (1:1000), p-EGFR (1:600), AKT (1:1000), p-AKT (1:800), ERK1/2 (1:1000), p-ERK1/2 (1:1000), α-tubulin (1:1000), H3K4me1 (1:1000), H3K4me2 (1:1000), or histone H3 (1:2000), respectively, overnight at 4 °C. Immunodetection was achieved after incubation with the corresponding secondary antibodies (1:10,000) in TBS-T for 1 h at RT. ECL reagents were used to reveal the positive bands on the membrane. To perform densitometry analysis, digital images of the positive bands were obtained with ChemiDoc XRS and analyzed using the image analysis program Quantity One (Bio-Rad). The results were presented as the target protein/loading control ratio.

### Cell migration assay

For the wound-healing assay, cells were seeded in a 6-well plate and allowed to grow until reaching 100% confluence. Mytomycin C (10 μg/ml) was introduced 2 h prior to the beginning of the assay to inhibit cell proliferation. A wound was then generated by scratching a straight line using a 10-μl pipette tip. The cells were washed twice and then cultured in serum-free medium for 24 h. The migration of the cells into denuded areas was monitored and visualized using a phase contrast microscope (40 × magnification). Accurate wound measurements were performed at 0 and 24 h to calculate the migration rate according to the following formula: percentage of wound healing = [(wound length at 0 h) − (wound length at 24 h)]/(wound length at 0 h) × 100. At least three independent experiments were performed.

For the migration assay, 1.5 × 10^5^ cells in 300 μl of serum-free medium were placed in the upper chamber of a Transwell system (BD Falcon^TM^ Cell Culture Inserts, BD Biosciences, Bedford, MA, USA). The chamber was then transferred to a 24-well culture plate containing 500 μl of medium with 10% FBS. After incubation for 24 h at 37 °C, the cells on the upper surface of the membrane (8-μm pore size) were removed with a wet cotton swab. The cells on the lower surface of the membrane were fixed with ice-cold methanol for 10 min and then stained in Giemsa solution for 15 min. The stained cells were subjected to microscopic examination under a light microscope. The migrated cells were counted in five randomly selected fields (100 × magnification) within each membrane, and the values were averaged. All experiments were performed with three replicates under each set of migration conditions.

### Statistical analysis

All values are presented as the mean ± SEM. The data were analyzed using Student’s *t-*test or one-way ANOVA with SPSS 11.5 software (SPSS Inc.). *p* values with a 95% confidence interval were obtained from at least three independent experiments. A *p* value of less than 0.05 was considered statistically significant.

## Additional Information

**How to cite this article**: Shao, G. *et al.* Lysine-specific demethylase 1 mediates epidermal growth factor signaling to promote cell migration in ovarian cancer cells. *Sci. Rep.* **5**, 15344; doi: 10.1038/srep15344 (2015).

## Supplementary Material

Supplementary Information
