# Supplementary material for: Lysine-specific demethylase 1 mediates epidermal growth factor signaling to promote cell migration in ovarian cancer cells
Source: Sci Rep. 2015 Oct 22;5:15344. doi: 10.1038/srep15344 (PMC4614681; doi:10.1038/srep15344)
Supplement: Supplementary Information [file srep15344-s1.pdf]

Supplementary Information

Lysine-specific demethylase 1 mediates epidermal growth factor signaling to promote cell migration in ovarian cancer cells

Genbao Shao[1,*], Jie Wang[2,5], Yuanxia Li[1], Xiuwen Liu[1], Xiaodong Xie[4], Xiaolei Wan[2], Meina Yan[1], Jie Jin[1], Qiong Lin[1], Haitao Zhu[4], Liuping Zhang[1], Aihua Gong[1], Qixiang Shao[1,3], and Chaoyang Wu[2,*]

[1]School of Medicine, Jiangsu University, Zhenjiang 212013, Jiangsu, P. R. China

[2]Department of Oncology, the Affiliated People's Hospital, Jiangsu University, Zhenjiang 212002, Jiangsu, P. R. China

[3]Jiangsu Key Laboratory of Medical Science and Laboratory Medicine, School of Medicine, Jiangsu University, Zhenjiang 212013, Jiangsu, P. R. China

[4]Department of Radiology, the Affiliated Hospital, Jiangsu University, Zhenjiang 212001, Jiangsu, P. R. China

[5]Gaochun People's Hospital, Nanjing 211300, Jiangsu, P. R. China

[*]Corresponding authors, e-mail: gbshao07@ujs.edu.cn and wuchaoyang9@163.com

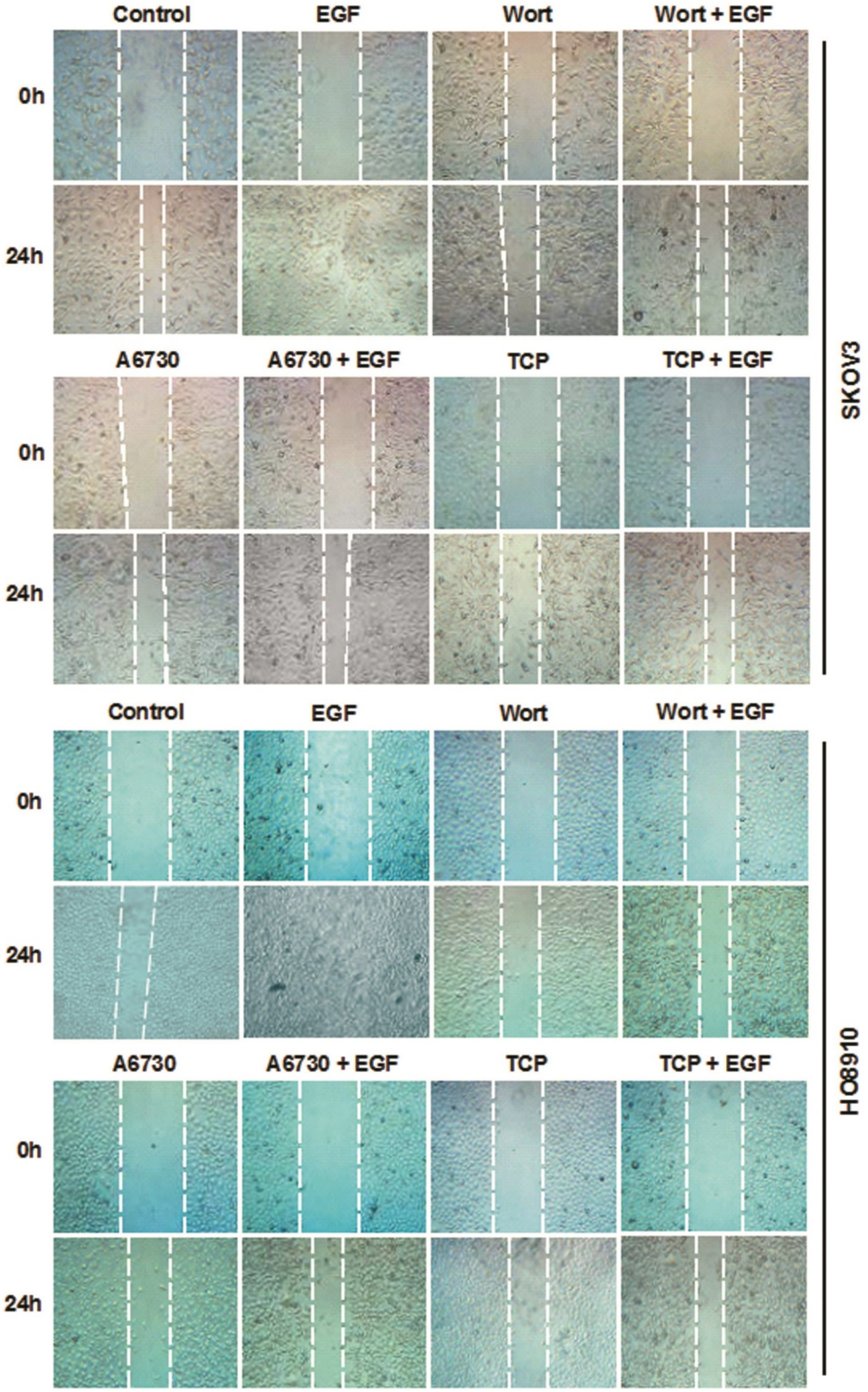

Figure S1 In the wound-healing assays, SKOV3 and HO8910 cells were pretreated

with wortmannin (1 μM), A6730 (20 μM) or TCP (100 μM) for 30 min and then stimulated with 100 ng/ml EGF in the presence of the inhibitors for 24 h. Representative images obtained at 0 and 24 h show repopulation of the wounded areas of untreated and treated cells (40× magnification).

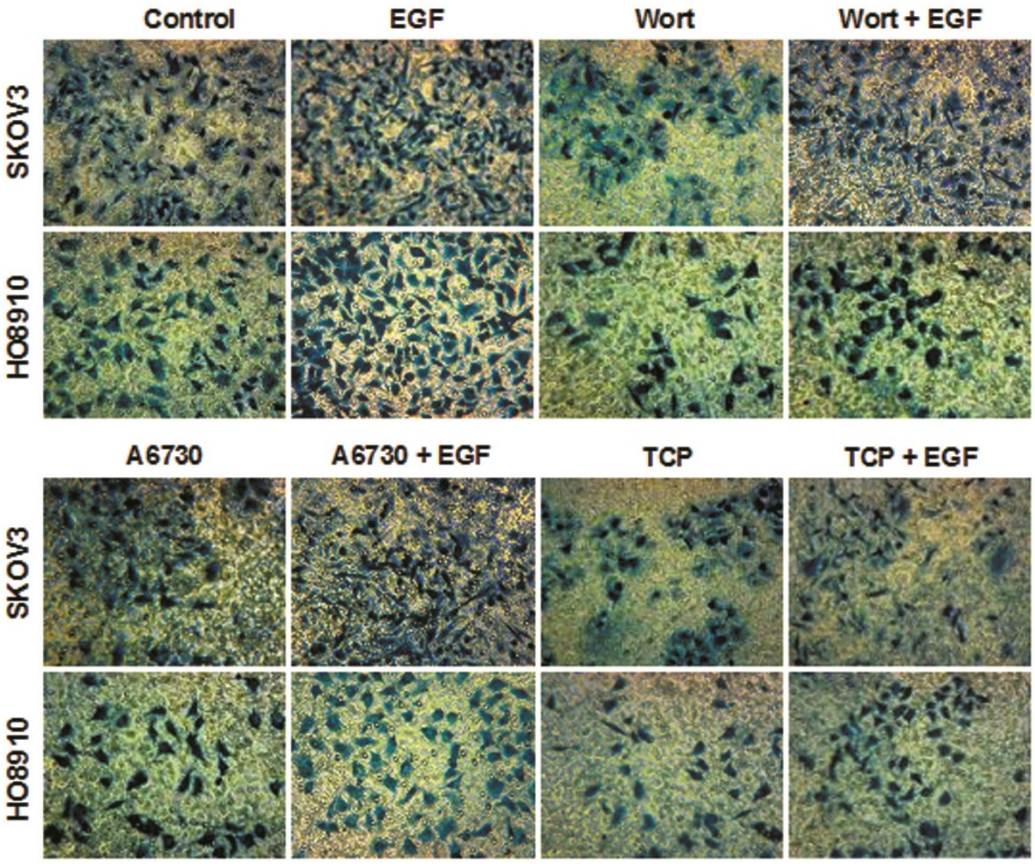

Figure S2 In the Transwell migration assays, SKOV3 and HO8910 cells were pretreated with wortmannin (1 μM), A6730 (20 μM), or TCP (100 μM) for 30 min and then seeded in Transwell inserts and cultured with 100 ng/ml EGF in the presence of the inhibitors for 24 h. Microphotographs from 24 h show representative fields of the Giemsa-stained lower membranes of the Boyden chambers (400× magnification).
